# T cell infiltration into Ewing sarcomas is associated with local expression of immune-inhibitory HLA-G

**DOI:** 10.18632/oncotarget.23815

**Published:** 2017-12-22

**Authors:** Christian Spurny, Sareetha Kailayangiri, Bianca Altvater, Silke Jamitzky, Wolfgang Hartmann, Eva Wardelmann, Andreas Ranft, Uta Dirksen, Susanne Amler, Jendrik Hardes, Maike Fluegge, Jutta Meltzer, Nicole Farwick, Lea Greune, Claudia Rossig

**Affiliations:** ^1^ Department of Pediatric Hematology and Oncology, University Children´s Hospital Muenster, Muenster, Germany; ^2^ Gerhard Domagk Institute of Pathology, University of Muenster, Muenster, Germany; ^3^ University Hospital Essen, Pediatrics III, West German Cancer Centre, Essen, Germany; ^4^ Institute of Biostatistics and Clinical Research, University of Muenster, Muenster, Germany; ^5^ Department of Orthopedic Surgery, University Hospital Muenster, Muenster, Germany; ^6^ Cells-in-Motion Cluster of Excellence (EXC 1003 – CiM), University of Muenster, Germany

**Keywords:** Ewing sarcoma, HLA-G, T cells, cellular immunotherapy, immune checkpoints

## Abstract

Ewing sarcoma (EwS) is an aggressive mesenchymal cancer of bones or soft tissues. The mechanisms by which this cancer interacts with the host immune system to induce tolerance are not well understood. We hypothesized that the non-classical, immune-inhibitory HLA-molecule HLA-G contributes to immune escape of EwS. While HLA-G^pos^ suppressor T cells were not increased in the peripheral blood of EwS patients, HLA-G was locally expressed on the tumor cells and/or on infiltrating lymphocytes in 16 of 47 pretherapeutic tumor biopsies and in 4 of 12 relapse tumors. HLA-G expression was not associated with risk-related patient variables or response to standard chemotherapy, but with significantly increased numbers of tumor-infiltrating CD3+ T cells compared to HLA-G^neg^ EwS biopsies. In a mouse model, EwS xenografts after adoptive therapy with tumor antigen-specific CAR T cells strongly expressed HLA-G whereas untreated control tumors were HLA-G^neg^. IFN-γ stimulation of EwS cell lines *in vitro* induced expression of HLA-G protein. We conclude that EwS cells respond to tumor-infiltrating T cells by upregulation of HLA-G, a candidate mediator of local immune escape. Strategies that modulate HLA-G expression in the tumor microenvironment may enhance the efficacy of cellular immunotherapeutics in this cancer.

## INTRODUCTION

Ewing sarcoma (EwS) is an aggressive malignancy with a characteristic age peak in adolescence. The outcome in patients with disseminated disease involving the bone and/or bone marrow remains poor, and attempts to eradicate residual disease by dose intensification of chemotherapy have failed to prevent fatal relapses in these patients [1, 2]. Since immune effector cells eliminate tumor cells by alternative mechanisms than cytotoxic drugs, T cells with native or engineered specificity for EwS-associated antigens may be potent tools to sustain remission after conventional therapies [3–6]. But despite their conceptual promise, immunotherapies have yet failed to make an impact on clinical outcome in this cancer. In a preclinical model, we found that T cells engineered to express chimeric antigen receptors (CAR T cells) against the EwS-associated surface antigen G_D2_ effectively interact with G_D2_-expressing EwS cells *in vitro*, yet their activity against tumor xenografts *in vivo* was limited [4, 7]. Also in other solid cancers [8, 9], the *in vivo* preclinical and early clinical efficacy of CAR T cell therapy has remained well below the expectations raised by the successful clinical trials in acute lymphoblastic leukemia [10–12]. A potential explanation is the presence of immune-inhibitory ligands and soluble agents in the microenvironment of solid tumors that tolerize T cells and render them dysfunctional against tumor targets (reviewed in [13, 14]). Identification of the mechanisms by which EwS cells manipulate local interactions with immune effector cells is a prerequisite for developing effective immunotherapeutic strategies.

Recently, the nonclassical MHC class I molecule HLA-G has emerged as an important regulator of immune responses and a potential mediator of cancer immune resistance. HLA-G is expressed on trophoblast cells during pregnancy where it has a physiological role in establishing immune tolerance at the maternal-fetal interface [15]. HLA-G is characterized by a limited polymorphism, with 7 isoforms (HLA-G1 to G7) that interact with three inhibitory receptors: KIR (killer cell immunoglobulin-like receptor) 2DL4, ILT (immunoglobulin-like transcript) 2, and ILT4. HLA-G has direct inhibitory effects on NK cells and T cells [15–18], and induces and expands myeloid suppressor cells [19]. Expression of HLA-G on T cells defines a subpopulation with potent suppressive function [20, 21]. There is substantial evidence that HLA-G can contribute to tumor immune evasion: HLA-G expression on tumor cells or secretion by bystander cells was found in various cancers and in some of these was associated with poor outcome [22–25]. *In vitro*, HLA-G expressed on tumor cells or soluble HLA-G inhibits antitumor NK cell and T cell responses [24–26]. In an immunocompetent mouse model, HLA-G alone was sufficient to prevent rejection of human tumors [19], illustrating the potency of HLA-G in mediating tumor immune escape.

Besides trophoblast cells, HLA-G is physiologically expressed in mesenchymal stroma cells (MSC) where it is associated with the high immunosuppressive potential of this cell population [27, 28]. The supposed tissue origin of EwS from MSC and the phenotypic and functional similarities with this cell type [29] led us to explore HLA-G as a candidate contributor to local immune suppression in EwS.

## RESULTS

### EwS patients do not have increased proportions of circulating HLA-G^pos^ suppressor T cells in peripheral blood

**Figure 1:**
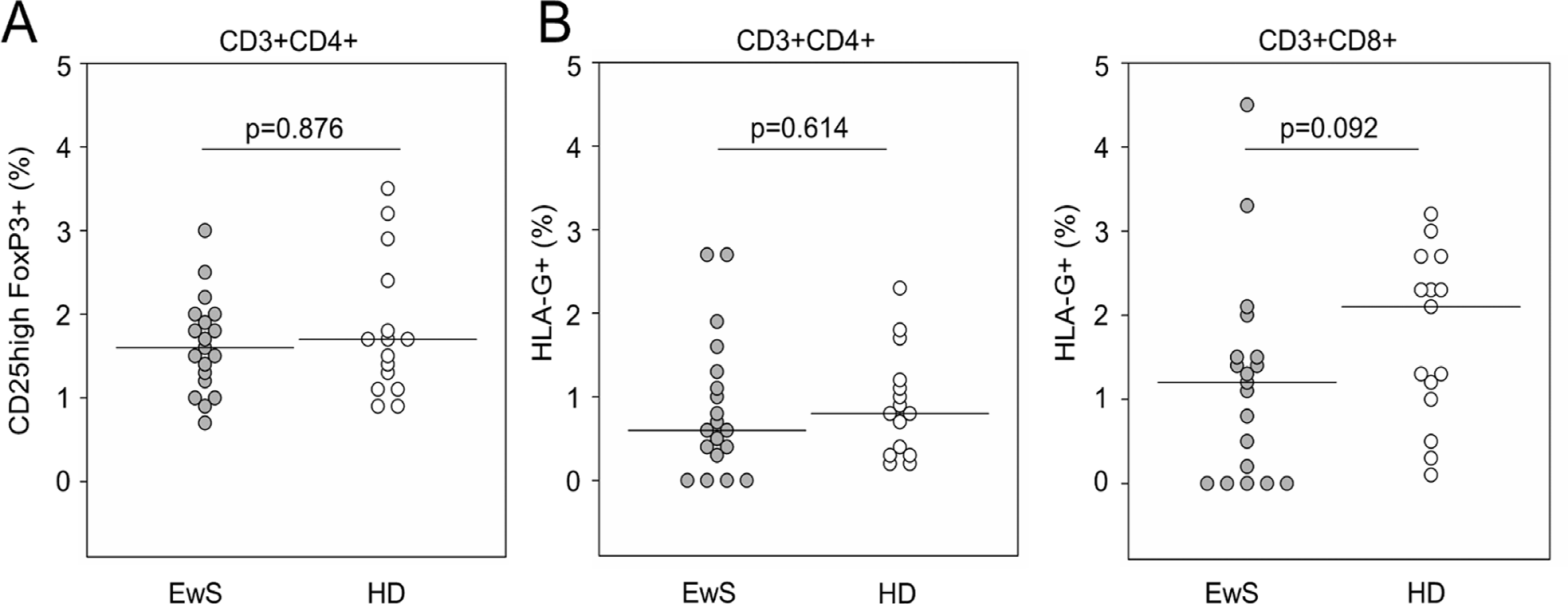
EwS patients do not have increased proportions of circulating HLA-G^pos^ T cells in peripheral blood Flow cytometry quantification of isolated PBMCs populations. Relative proportions of (**A**) FoxP3+ CD25^high^ Treg cells as a fraction of CD4+ T cells, and of (**B**) HLA-G^pos^ T cells as fractions of CD4+ (left panel) or CD8+ T cells (right panel) in 19 EwS patients and 15 healthy donors (HD). *P*-values were calculated using a Mann-Whitney test.

Circulating HLA-G^pos^ T cells have potent regulatory properties and can mediate systemic immune suppression [20]. We determined the proportions of HLA-G^pos^ T cells among peripheral blood (PB) CD4+ and CD8+ T cells, respectively, in 19 EwS patients and 15 healthy donors by flow cytometry. For reference, we compared the proportions of circulating T cells with the classical regulatory phenotype of FoxP3 expression along with CD25^high^ and CD4 (FoxP3^pos^ Treg cells). EwS patients and healthy donors had equal proportions of FoxP3^pos^ Treg cells (*p* = 0.876) (Figure 1A). The proportions of PB HLA-G^pos^ T cells were also not noticeably different between patients and healthy donors, neither among CD4+ T cells (median 0.6% (range 0.0 to 2.7%) versus median 0.8% (range 0.2 to 2.3%), *p* = 0.614) nor CD8+ T cells (median 1.2% (range 0.0-4.5%) versus median 2.1% (range 0.1 to 3.2%), p 0.092) (Figure 1B). Thus, EwS patients do not have increased proportions of HLA-G^pos^ T cells in PB.

### HLA-G is expressed in the local tumor microenvironment of newly diagnosed and relapsed Ewing sarcomas

**Figure 2:**
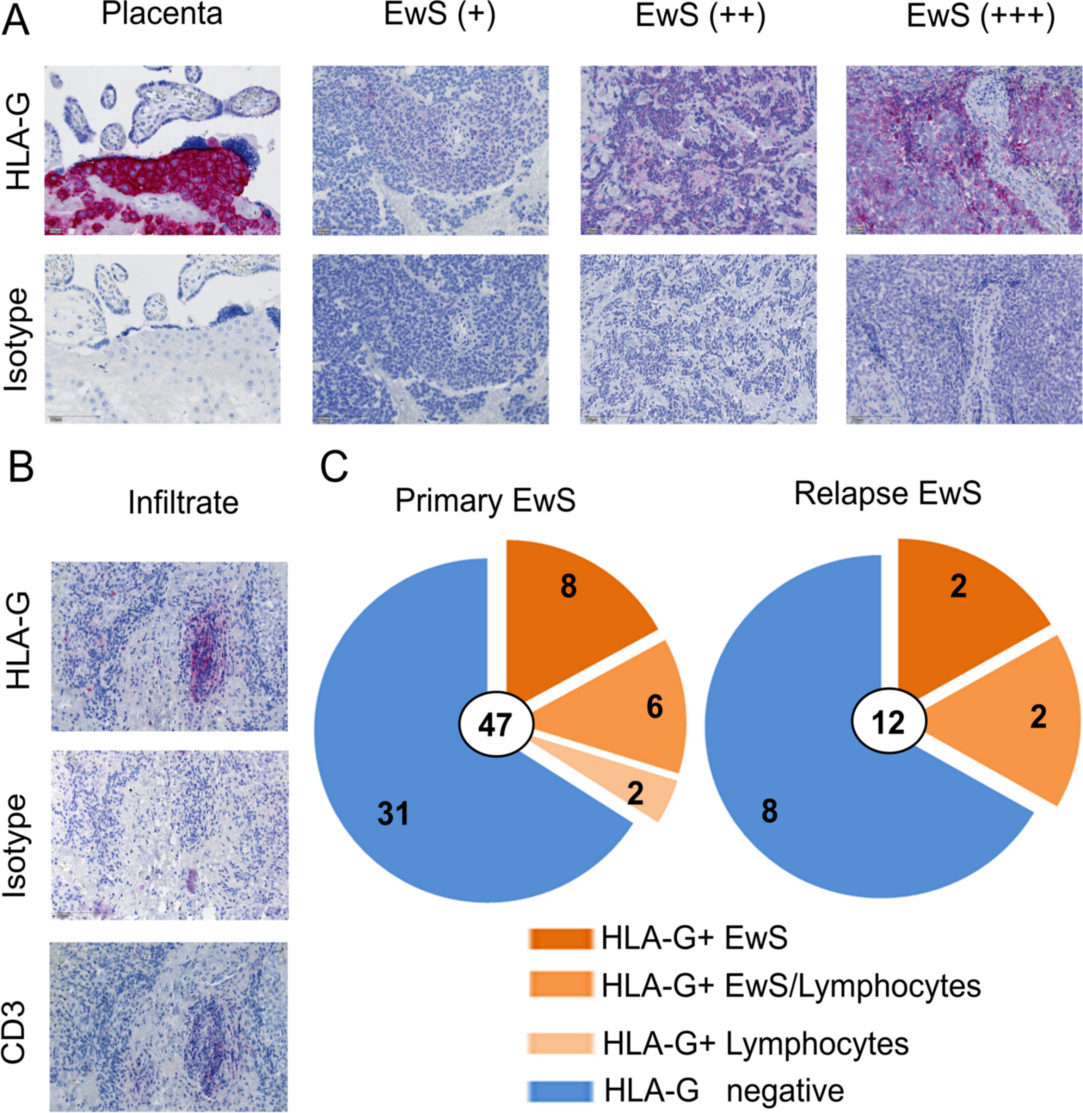
HLA-G is expressed in the tumor microenvironment of EwS patients (**A**) HLA-G expression detected by immunhistochemistry after staining of sections from paraffin embedded EwS biopsies with HLA-G specific antibody (4H84) or isotype control (MOPC-21). Human placenta tissue was used as positive control. Examples for weak (+), median (++), and strong (+++) expression are shown. (**B**) Example of HLA-G and CD3 expression on infiltrating lymphocytes in a EwS biopsy. (**C**) Summary of all tumor samples analyzed at primary diagnosis and at relapse. All biopsies were pre-therapeutic.

Next, we analyzed HLA-G expression in EwS biopsies. Paraffin-embedded pretherapeutic tumor biopsies from 49 pediatric and young adult patients with newly diagnosed (*n* = 47) and/or relapsed (*n* = 12) EwS were analyzed by immunohistochemistry using the HLA-G specific antibody clone 4H84. Patient characteristics are found in Table 1. Human placenta tissue, the main site of physiological HLA-G expression, was used as a positive control. HLA-G was found to be expressed at either low, intermediate or strong densities in 16 of the 47 treatment-naive EwS biopsies (34%), either on the tumor cells (14 of 47, 30%) (Figure 2A, 2C) and/or on infiltrating lymphocytes (8 of 47, 17%) (Figure 2B, 2C). In six samples, HLA-G was detected both on tumor cells and on infiltrating lymphocytes, whereas HLA-G expression exclusively on lymphocytes was found in two samples. HLA-G staining of EwS cells and bystander cells of the microenvironment was membraneous and cytoplasmic by light microscopy, nuclear stainings were not observed. HLA-G expression was typically focal, with varying proportions of HLA-G^pos^ tumor cells clustered in areas of the individual tumors. Among the 12 relapse samples, 4 (33%) expressed HLA-G on EwS cells, of which 2 also contained HLA-G^pos^ infiltrating lymphocytes. The analysis included 10 patients with samples obtained both at first diagnosis and at relapse, allowing for intraindividual comparisons of the two manifestations. Two patients had HLA-G^pos^ tumors both at diagnosis and at relapse, and 5 were HLA-G^neg^ at both time-points. In 1 patient, HLA-G^pos^ lymphocytes were identified in the tumor at first diagnosis, but not at relapse. In 2 patients with HLA-G^neg^ tumors at first diagnosis, relapse tumors expressed HLA-G. In an individual patient, we were able to study HLA-G expression both on her primary tumor located in the fibular bone and in an inguinal lymph node metastasis, and found HLA-G expressed at both sites. In 10 patients evaluable for both HLA-G expression in tumors and on cirulating T cells (Figure 1B), we did not find any association between the two parameters. Specifically, of three patients with HLA-G expressing lymphocytes in their tumors, two had HLA-G^pos^ circulating T cells in PB above the median, whereas the third patient did not have any detectable CD4+ or CD8+ HLA-G^pos^ T cells.

**Table 1:** Patient characteristics

	*n =* 47
Age	
median/range (years)	15.4 (2.5–44.4)
≤ 14 years	16
> 14 years	31
Gender	
Male	32
Female	15
Male-to-female ratio	2.13
Tumor site	
Axial	21
Extremity	24
unknown	2
Primary distant metastases	
Absent	28
Pulmonary only	10
Bone and/or bone marrow only	2
Pulmonary and bone and/or bone marrow	6
unknown	1
Tumor response	
Good (Grades 1–3)	28
Poor (Grades 4–6)	6
Missing^*^	13

^*^Information on tumor response was missing in patients with unresectable tumors after neoadjuvant chemotherapy who received radiotherapy as sole local therapy.

We conclude that HLA-G is expressed on tumor cells and in the tumor microenvironment in a substantial proportion of both primary and relapsed untreated EwS. HLA-G expression is not always consistent between primary and relapse manifestations. HLA-G expressing lymphocytes can be found in tumors even in the absence of circulating HLA-G^pos^ T cells, suggesting local induction of HLA-G expression.

### HLA-G expression in the EwS microenvironment is not associated with risk parameters and response to standard chemotherapy

To explore whether HLA-G expression in the tumor microenvironment is a feature of a specific subgroup of EwS, we studied potential associations with patient-related variables and previously described risk factors [2, 30] in patients with a first diagnosis of EwS. Neither age (≤ or >14 years) (*p* = 0.52), gender (*p* = 0.74), tumor site (extremity versus axis) (*p* = 0.76), tumor volume (≤ 200 ml or > 200 ml) (*p* = 0.52), nor the presence or absence of distant metastases in lungs, bone and/or bone marrow (*p* = 0.35) was significantly associated with HLA-G expression in the microenvironment of treatment-naive tumor samples. Moreover, with all the limitations of a small and retrospective analysis, no correlation of HLA-G expression with tumor response to neoadjuvant chemotherapy by Salzer Kuntschik grading was found (*p* = 0.64). All patients received standardized treatment within the multicenter clinical trials E.U.R.O Ewing 99 or Ewing 2008, with identical neoadjuvant chemotherapy, thus the response assessment was performed in a homogenously treated population of patients. The short follow-up of our patient cohort does not yet allow to assess a potential association of HLA-G positivity with event-free survival and outcome. We conclude that in our patient cohort HLA-G expression in the tumor microenvironment occurs independent of clinical parameters of the disease and response to standard cytotoxic agents.

### HLA-G expression in EwS is associated with T cell infiltration and is upregulated in response to tumor-antigen specific T cell therapy

**Figure 3:**
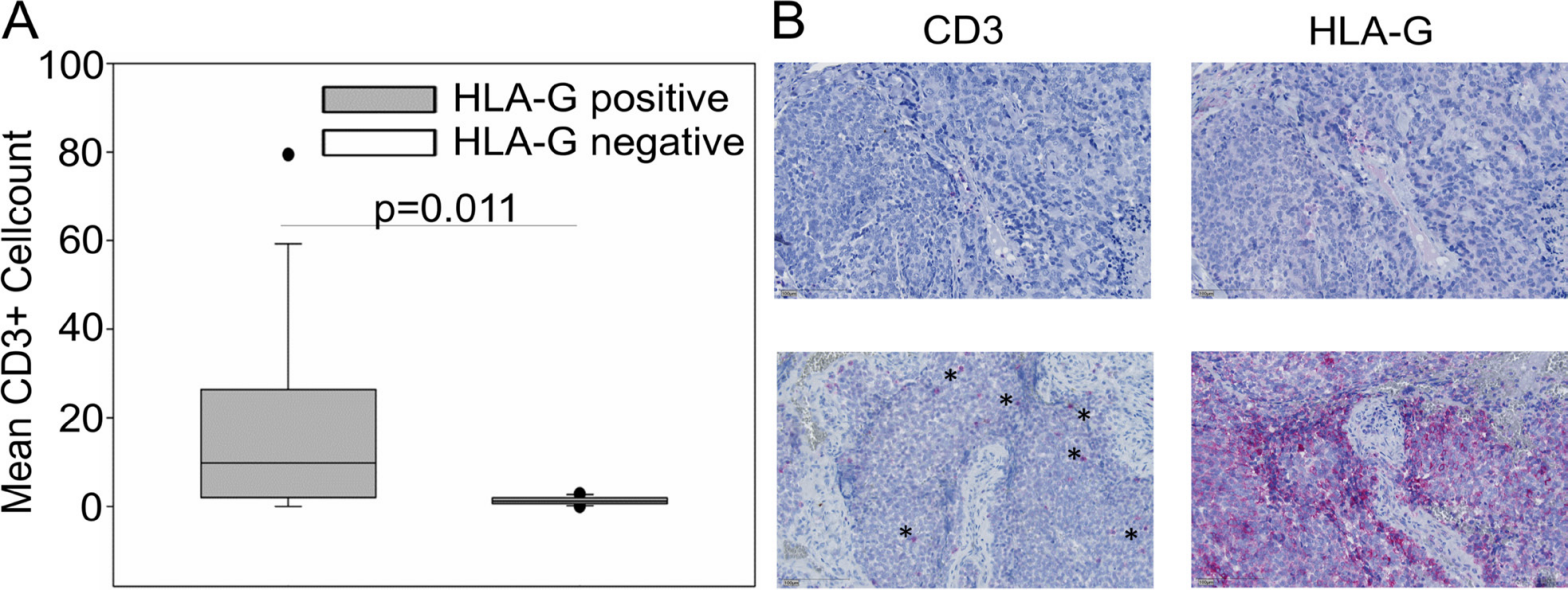
CD3+ T cell infiltration in HLA-G^pos^ EwS tumors is significantly enhanced compared to HLA-G^neg^ biopsies (**A**) CD3+ cells on 13 EwS biopsies from 11 patients corresponding to HLA-G positive tumors and 12 EwS biopsies corresponding to HLA-G negative tumors were detected by immunohistochemistry on serial tissue slides. Numbers of T cells were calculated as means of counted CD3+ cells from 4-5 high power fields (HPF). Statistical analysis was performed with the Mann-Whitney rank-sum test. (**B**) Examples of a biopsy with undetectable CD3+ cells and HLA-G^neg^ tumor cells (upper panel), and a biopsy with high CD3+ infiltrating cells (asterisks) and corresponding HLA-G^pos^ tumor cells (lower panel).

We hypothesized that HLA-G expression in EwS is a consequence of an inflammatory immune response in the local tumor microenvironment. To investigate whether local HLA-G expression in EwS is associated with the presence of immune cells, we compared numbers of infiltrating T cells in HLA-G^pos^ and HLA-G^neg^ EwS by immunohistochemistry. This analysis included 13 HLA-G^pos^ tumors samples (from 11 patients) that met the criteria for a quantitative analysis (see Methods), and 12 HLA-G^neg^ tumors. All in all, numbers of infiltrating T cells in EwS biopsies were rare. Significantly higher proportions of infiltrating CD3+ T cells were detected in HLA-G^pos^ compared to HLA-G^neg^ EwS biopsies (Figure 3). These data support our hypothesis that interactions between tumor cells and infiltrating T cells lead to upregulation of HLA-G.

**Figure 4:**
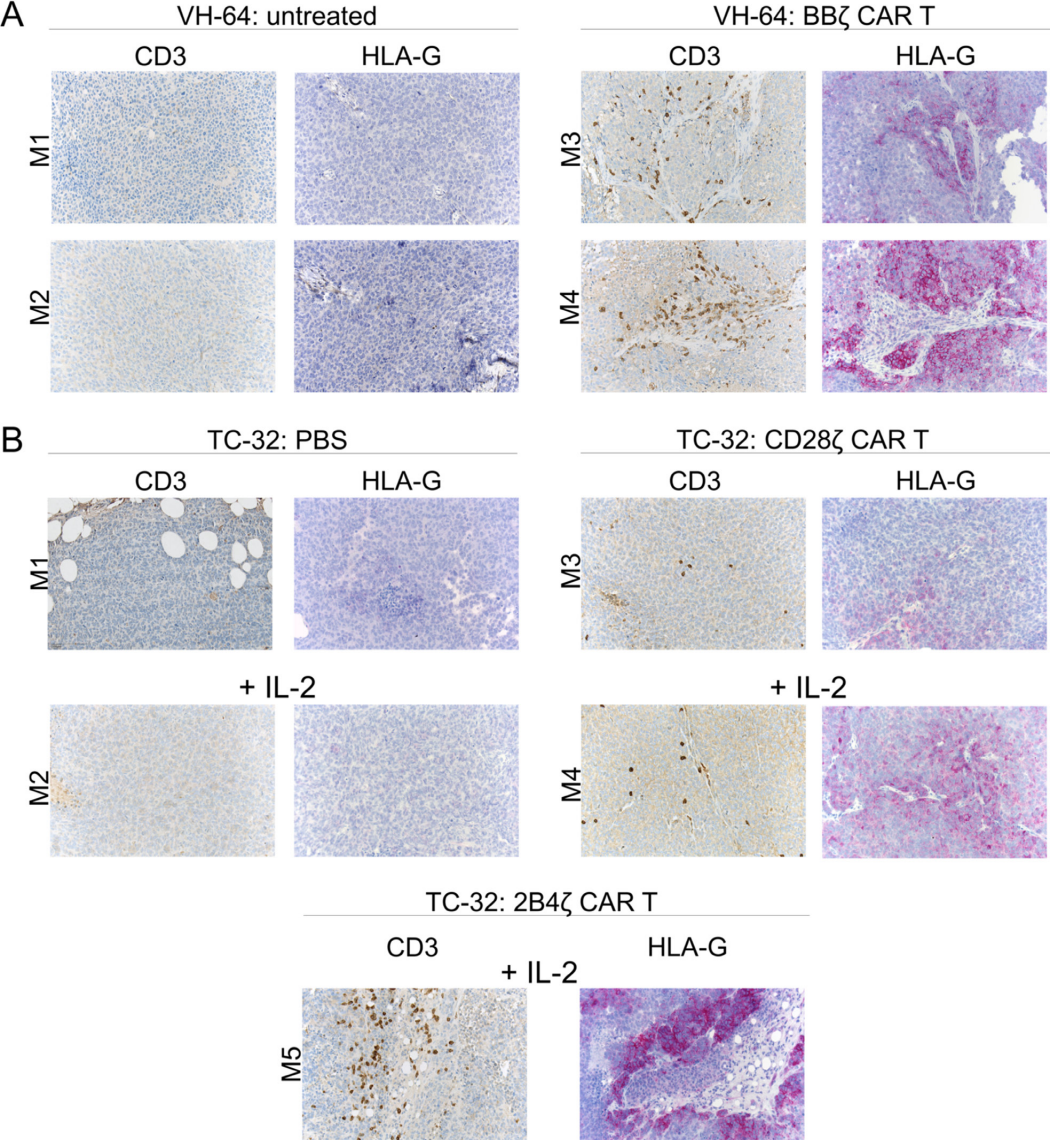
HLA-G expression in EwS xenografts following adoptive CAR T cell therapy (**A**) Expression of CD3 and HLA-G in FFPE tumor tissue sections obtained from mice either untreated (left panel) or on day 15 post therapy with GD2-BBζ-transduced T cells (right panel). (**B**) Expression of CD3 and HLA-G in FFPE tumor tissue sections obtained from mice receiving either PBS alone or with IL-2 (upper left panel) or treated with T cells transduced with a G_D2_-specific 28ζ CAR without and with IL-2 (upper right panel), or with a G_D2_-specific 2B4ζ CAR without and with IL-2 (lower panel). Shown is one tumor section of each mouse treated.

To obtain direct support for this mechanism, we analyzed HLA-G expression on archived EwS xenografts following *in vivo* treatment with G_D2_-specific CAR T cells. In our first *in vivo* xenograft model, NSG mice had received 2 × 10^6^ VH-64 cells by s.c. administration, and palpable tumors had been treated with 3 i.v. doses of 1 × 10^7^ human T cells engineered to express a 2nd generation CAR, GD2-BBζ, directed against ganglioside G_D2_, which is expressed at high densities on VH-64 cells [4]. No cytokines had been given to the mice. HLA-G expression on tumor cells was determined by immunohistochemistry upon autopsy. Tumors growing in two mice who had received GD2-BBζ CAR T cells but not tumors in two untreated control animals strongly expressed HLA-G (Figure 4A). Infiltrating human T cells were detected in the tumors of both mice after T cell transfer (Figure 4A).

We reproduced this finding with archived tumor samples from additional experiments which had investigated T cell therapy using two other G_D2_-specific 2nd generation CARs with alternative costimulatory domains, either CD28 or 2B4 [31]. Mice with i.p. xenografts of the G_D2_+ EwS cell line TC-32 had received either PBS alone or combined with IL-2, or four consecutive injections of CAR T cells along with or without IL-2 by i.p. administration. Again we detected infiltrating T cells in all tumors following adoptive CAR T cell transfer (Figure 4B). As in VH-64 xenografts and with the use of a 4-1BB CAR, G_D2_-specific CAR T cell therapy was associated with HLA-G expression in all xenografts, whereas controls not receiving T cells were HLA-G^neg^ (Figure 4B). Notably, IL-2 administration alone was not associated with HLA-G expression. We conclude that tumor infiltration with T cells in EwS is associated with upregulation of HLA-G.

### EwS cell lines can respond to IFN-γ cytokine stimulation with expression of HLA-G

**Figure 5:**
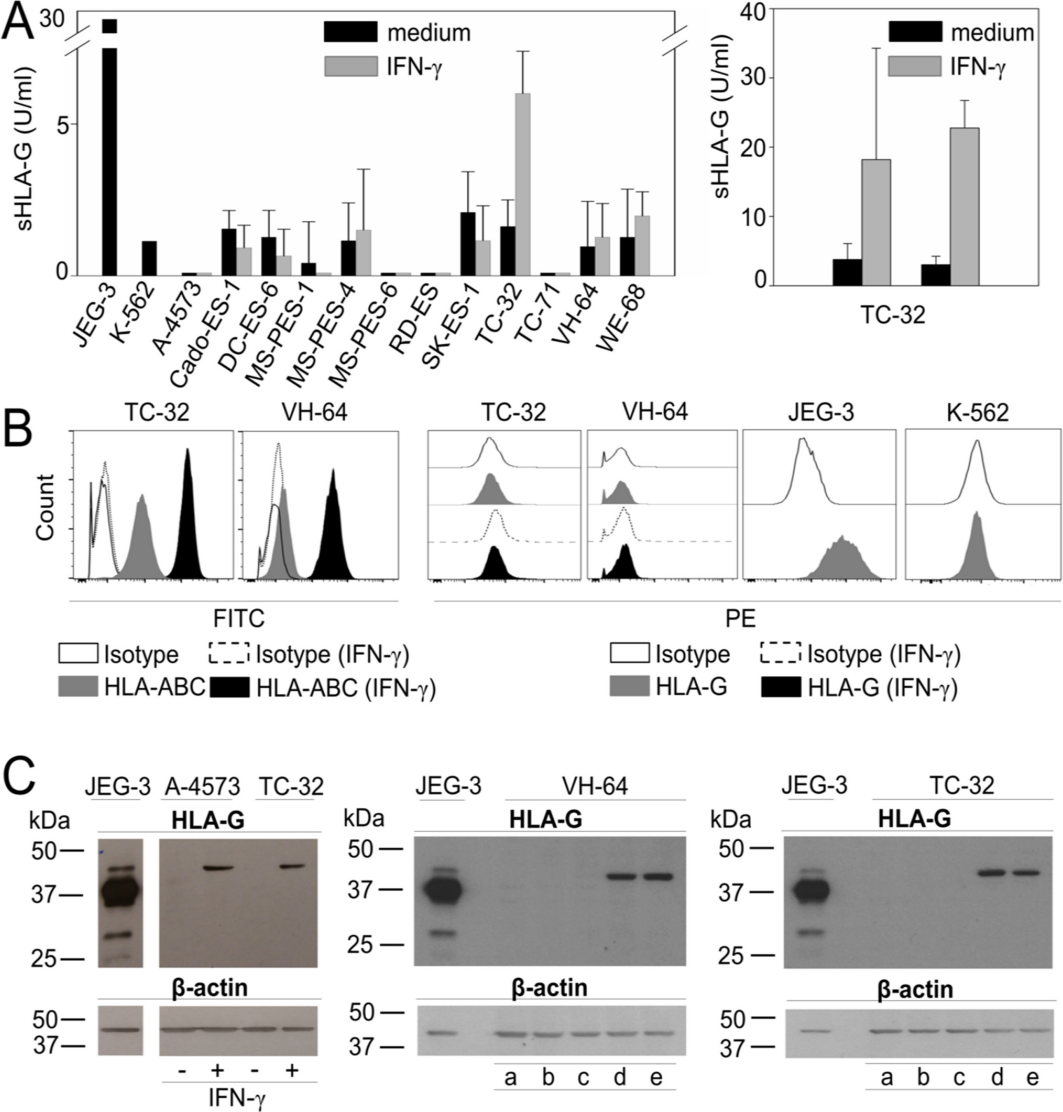
HLA-G is expressed in EwS cell lines in response to stimulation with IFN-γ and in the presence of CAR T cells (**A**) Single screen of soluble HLA-G in 12 EwS cell culture supernatants by ELISA with and without stimulation with 500 U/ml IFN-γ for 48 hours (left panel) and reproduction of soluble HLA-G detection in TC-32 supernatants with and without IFN-γ for 48 hours (right panel) (two experiments). (**B**) HLA-ABC and HLA-G expression in EwS cells with and without stimulation with 500 U/ml IFN-γ for 48 hours by flow cytometry. Shown are examples of two EwS cell lines VH-64 and TC-32. JEG-3 was used as positive control and K-562 as HLA-G negative control (two experiments) (**C**) HLA-G protein detected by Western Blot analysis with HLA-G specific clone 4H84 in EwS cell lines A-4573 and TC-32 with and without IFN-γ stimulation (left panel), and in VH-64 (central panel) and TC-32 (right panel) after incubation with medium (a), conditioned medium from cultures of non-transduced (NT) alone (b) and cocultured with EwS cells (c), or from GD2-BBζ CAR T cells alone (d) alone and cocultured with EwS cells (e) (two experiments). JEG-3 was used as positive control.

Next we addressed the mechanism by which T cell infiltration leads to HLA-G expression in EwS. To investigate whether the inflammatory T cell cytokine IFN-γ can induce HLA-G expression in EwS cells, we quantified HLA-G isoforms in cell culture supernatants by ELISA using MEM-G/9 mAb which detects the soluble isoforms HLA-G1 and HLA-G5. Without pretreatment, neither of the EwS cell lines secreted HLA-G isoforms above the level of the HLA-G^neg^ control cell line, K-562. In one cell line, TC-32, IFN-γ pretreatment induced secretion of HLA-G1/G5 (Figure 5A), proving principle that at least individual Ewing sarcomas can secrete soluble HLA-G under inflammatory conditions.

Next we explored whether EwS cells can express surface-bound HLA-G isoforms. We analyzed 14 EwS cell lines by flow cytometry, using PE-labeled anti-HLA-G1 antibody clone MEM-G/9. Surface HLA-ABC was stained for comparisons. Whereas all cell lines expressed at least low densities of classical HLA molecules ABC, and pretreatment with the inflammatory cytokine IFN-γ consistently increased HLA-ABC expression, HLA-G1 was not detected on the cell surface of any of the EwS cell lines, and IFN-γ stimulation failed to induce HLA-G expression (Figure 5B, Supplementary Figure 1). MEM-G/9 detects HLA-G1 only in association with ß2-microglobulin whereas mAb 4H84, used for immunohistochemistry detection of HLA-G in Figure 2, recognizes all denatured isoforms. Since 4H84 is not validated for flow cytometry, we used Western Blot analysis to detect HLA-G in protein lysates of A-4573 and TC-32 EwS cell lines after IFN-γ stimulation. Neither A-4573 and TC-32, nor two additional EwS cell lines, VH-64 and DC-ES-6 constitutively express HLA-G protein, but all 4 cell lines upregulate HLA-G upon IFN-γ stimulation (Figure 5C, and [31]). Thus, EwS cells respond to IFN-γ cytokine stimulation with expression of HLA-G protein.

Finally, we mimicked the interaction between T cells and EwS cells by adding supernatants from cultures of unmodified T cells and G_D2_-specific CAR T cells, or cocultures of either type of T cells with G_D2_-positive EwS cells, to EwS cell cultures. HLA-G protein expression in EwS cells were analyzed by Western Blot analysis. Indeed, conditioned medium from culture and coculture supernatants of CAR T cells were able to induce HLA-G protein expression in EwS cells (Figure 5C). Thus, soluble factors released by CAR T cells can mediate HLA-G upregulation by the tumor cells. We conclude that EwS can respond to immune targeting by upregulation of HLA-G, and that this response is at least partially cytokine-mediated.

## DISCUSSION

Mechanisms by which cancer cells tolerize infiltrating immune effector cells and prevent effective anti-tumor immune responses have central roles in both local and metastatic tumor growth and in resistance to current cancer therapies. Identification of specific immune-inhibitory pathways in the cancer microenvironment has led to effective interventions. Impressive clinical responses have been obtained by therapeutic blockade of the immune-inhibitory receptor PD-1 or its ligand in individual cancers, including metastatic melanoma [32], non-small cell lung cancer [33] and Hodgkin lymphoma [34], but not in many others. The association of clinical responses to PD-1 pathway blockade with a high mutational burden [35], PD-L1 expression in tumors [36], and the presence of infiltrating CD8+ T cells [37], opposed to the lack of all these features in EwS [38, 39] (and Figure 3) argues against a major clinical impact of PD-L1/PD-1 blockade in this cancer. Alternative mechanisms may exist by which EwS avoid infiltration with T cells and other immune effector cells and local immune activation. Here we obtained evidence for a contribution of HLA-G to the non-immunogenic nature of EwS.

We detected HLA-G on tumor cells as well as on infiltrating lymphocytes in a substantial proportion of about one third of EwS biopsies both at first manifestation and at relapse. A potential explanation for the limited detection of HLA-G in only a proportion of EwS is that HLA-G expression in tumor tissues may be a dynamic event and may vary between different areas in individual tumors. Even though we used large tumor biopsies obtained by open surgery, HLA-G^pos^ areas in individual tumors may have been overlooked, resulting in an underestimation of the true proportion of HLA-G-expressing EwS. Intratumoral heterogeneity could also explain the inconsistency of HLA-G expression at first diagnosis and at relapse in some of the serial biopsies. The observation that HLA-G was expressed at both of two tumor sites in one patient supports uniformity of this feature but remains anecdotal and is difficult to reproduce since routine diagnostic procedures do not stipulate pretherapeutic biopsies from more than one site.

Importantly, whereas HLA-G^neg^ EwS were only scarcely infiltrated with T cells, HLA-G expression was associated with the presence of T cells in the tumor microenvironment, suggesting a causal link between T cell infiltration and upregulation of HLA-G. Local induction of HLA-G in the presence of inflammatory T cell infiltrates has also been reported in melanoma [40]. Here we further obtained direct evidence that EwS can respond to infiltration with T cells by upregulation of HLA-G: CAR T cell therapy of tumor xenografts *in vivo* was also associated with HLA-G expression. Although the experiment was limited by the availability of archived tumor samples from only low numbers of mice and the lack of control animals treated with non-transduced T cells, it clearly demonstrates that infiltrating CAR T cells compared to IL-2 stimulation alone, irrespective of the type of costimulation provided within the CAR, induce HLA-G expression. This is in agreement with our recent finding that HLA-G is upregulated in EwS xenografts also in response to treatment with activated and G_D2_ CAR gene-modified NK cells [31]. Thus, local expression of HLA-G in EwS is a robust response to cellular immune targeting reproducible across both clinical and several experimental conditions.

We have started to elucidate the underlying mechanisms and found that both the cytokine IFN-γ and supernatants from CAR T cell cultures, alone and in cocultures with CAR antigen-expressing target cells, are inducers of HLA-G protein expression in EwS cells. This suggests that HLA-G is upregulated in EwS as a consequence of stimulation with inflammatory cytokines released by immune effector cells within the tumor microenvironment. Our failure to detect HLA-G expression by flow cytometry may be explained by the restricted binding capacity of the antibody clone MEM-G/9 validated for flow cytometry which detects only one of the surface-bound HLA-G isoforms and only in association with β2-microglobulin and may have missed alternative isoforms expressed in EwS. Alternatively, HLA-G may be limited to shed or secreted isoforms not expressed on the tumor cell surface, as shown in neuroblastoma [24]. The type and localization of HLA-G expressed in the EwS microenvironment is critical to determine its significance in this cancer and to develop HLA-G blocking strategies. To differentiate between cytoplasmic and membraneous staining, we are currently performing confocal microscopy studies. We further aim to identify the specific HLA-G isoform(s) induced in EwS cells by mass spectrometry.

The key question is whether HLA-G expression in EwS is relevant to protect the tumor from natural and therapeutic immune responses. HLA-G has been found to be an independent predictive factor for poor outcome at least in some cancers [22]. In our patient cohort, HLA-G expression was not associated with high-risk parameters of the disease, nor with poor response to cytotoxic drugs. While this argues against a predominant biological role in natural disease progression and dissemination, these data will have to be validated in a large prospective cohort of uniformly treated patients with EwS with longer follow-up. Moreover, the statistical analysis may have been flawed by the intratumoral heterogeneity of HLA-G expression and the resulting sampling issues, as explained above. Finally, the highly intensive standard chemotherapy may have overridden potentially tumorprotective effects of HLA-G which can become relevant in the context of future immune-targeted therapies.

Important insights are likely to be gained by studying the functional effects of HLA-G expressed by tumor cells on the infiltrating lymphocytes. HLA-G can induce upregulation of the HLA-G receptor CD85j (ILT2) in T cells and NK cells [41], directly reduce cytolysis by T cells [42], induce apoptosis through CD8 ligation by soluble isoforms [43], or indirectly tolerize T cells by inducing populations of bystander cells with immune-suppressive function [24, 44]. Long et al. recently showed that xenografts from pediatric sarcomas contain high proportions of myeloid-derived cells capable to suppress CAR T cell responses *in vitro* [45]. Further expansion of these and other immune-inhibitory cells by upregulated HLA-G could contribute to immune escape of EwS from T cell targeting. The complexity of these mechanisms requires to study the phenotype and function of infiltrating T cells in the presence of HLA-G in the human tumor microenvironment, e.g. of humanized mouse models.

From the translational point of view, HLA-G could be an interesting therapeutic target. EwS with its low somatic mutation rate and limited number of infiltrating T cells is the prototype of an “immunological desert” [46]. Therefore it is highly unlikely that strong adaptive anti-tumor immune responses preexist in this cancer which are inhibited by HLA-G and/or other immune-inhibitory mechanisms and just need to be unleashed to reject the cancer. Still, HLA-G upregulation may become relevant when attempting to target the cancer using adoptive transfer of T cells [4–6], NK cells [31, 47] or active immunization [3]. The HLA-G barrier is highly robust, since it effectively overcomes alloimmunity to provide selective maternal-fetal immune tolerance. In a mouse model, HLA-G alone was able to prevent tumor immune rejection [19]. *In vitro*, HLA-G expression even in a minor subpopulation of tumor cells was sufficient to provide bystander protection of HLA-G^neg^ cells, though by unknown mechanisms [25]. In a previous study from our own group, HLA-G expression in EwS xenografts was associated with continued tumor growth despite treatment with activated NK cells expressing G_D2_-specific CARs [31]. Although various further mechanisms may have prevented efficacy, upregulation of HLA-G as a potential hurdle to adoptive cell therapy deserves further exploration. Prospective animal experiments that take into account the limitations of the xenograft model will aim to demonstrate the relevance of HLA-G in counteracting CAR-mediated antitumor activity by antibody blockade or by gene-editing of HLA-G expression. Specific interventions that bypass HLA-G mediated immune escape, e.g. blocking antibodies against the HLA-G isoforms upregulated in EwS, may substantially improve efficacy of cellular therapies in this and other cancers.

## MATERIALS AND METHODS

### Patient material

For peripheral blood (PB) analysis, blood samples were obtained from 15 healthy donors between 15 and 28 years of age, and from 19 patients with a first diagnosis of EwS prior to starting therapy. Mononuclear cells (MCs) were isolated from PB and BM by density gradient centrifugation, resuspended in RPMI culture medium (Invitrogen, Darmstadt, Germany), and frozen in liquid nitrogen or used directly. For immunohistochemistry analysis, paraffin-embedded pretherapeutic tumor biopsies were obtained from 47 pediatric and young adult patients with newly diagnosed Ewing sarcomas. From 10 of these patients, tumor biopsies were also available at relapse, and two additional biopsies were obtained at relapse alone. All patients were included into the multicenter E.U.R.O Ewing 99 and Ewing 2008 trials. The study was approved by the University of Muenster Ethical Board, and informed consent was obtained from donors, patients and/or their legal guardians in accordance with the Declaration of Helsinki. Detailed patient characteristics are found in Table 1.

### Cell culture

The following EwS cell lines were used: VH-64 and WE-68 were a gift from Frans van Valen´s laboratory at the Institute of Experimental Orthopedics of University of Muenster, Germany). A4573, 5838, TTC-466 and TC-32 were kindly provided by the Children’s Hospital Los Angeles. RD-ES, TC-71, SK-ES1 and CADO-ES-1 were obtained from DSMZ (Braunschweig, Germany). MS-PES-1, MS-PES-4, MS-PES-6, and DC-ES-6 were established in our lab from biopsy material obtained upon metastatic relapse. K562 is a human leukemia cell line, and JEG-3 is a human placental choriocarcinoma cell line (both from DSMZ). All tumor cells were cultured in RPMI 1640 medium, supplemented with 10% heat-inactivated fetal calf serum (FCS; Thermo Fisher, Bonn, Germany) and 2 mM L-glutamine (PAA, Cölbe, Germany), and maintained at 37°C and 5% CO_2_. EwS cell lines TC-32, TC-71, and 5838 were cultured in uncoated flasks, and all other EwS cell lines were cultured in collagen-coated 25 cm^2^ tissue culture flasks. The identity of the cell lines was confirmed by short tandem repeat (STR) profiling. For some experiments, EwS cell cultures with 30-50% confluence were stimulated with medium containing 500 U/ml IFN-γ for 48 hours.

### Flow cytometry

T cell phenotypes were determined using fluorescence-conjugated monoclonal antibodies (mAbs) against CD3 (clone SK7), CD8 (clone RPA-T8), and CD4 (clone SK3) (all from BD, Heidelberg, Germany). Cell surface expression of membrane-bound HLA-G1 was analyzed with PE-labeled antibody clone MEM-G/9 (Exbio, 1P-292-C100), and HLA-ABC molecules were detected using FITC-labeled antibody clone W6/32 (Serotec, MCA81F). For each sample a minimum of 10,000 cells was analyzed with FACS Canto and FACS Diva Software. Intracellular FoxP3-expression was analyzed with the anti-human FoxP3 staining kit according to the manufacturers recommendations (eBioscience, San Diego, CA, USA). The percentage of CD25^hi^/FoxP3-positive cells was determined after gating on CD3+/CD4+ cells.

### Immunohistochemistry and CD3 quantification

Tumor samples were routinely obtained by open surgical biopsy. Formalin-fixed paraffin-embedded (FFPE) tumor sections were stained with the HLA-G specific mAb clone 4H84 (Exbio, 11-499-C100), which detects all HLA-G isoforms using Autostainer (Dako, 3400-9580-03). Biotinylated secondary antibodies were visualized with red chromogen or brown 3,3’-diaminobenzidine tetrahydrochloride (DAB) for HLA-G or CD3 according to the manufacturer (Dako K5001, K5005). CD3 quantification (Thermo Scientific, RM-9107-S1) was done on serial tissue sections corresponding to HLA-G^pos/neg^ tumor biopsies, along with expert histomorphology review by an experienced pathologist. Cytomorphological features, particularl nuclear size and shape as well as chromatin quality were thoroughly assessed to differentiated between (larger and atypical) EwS cells and infiltrating lymphocytes. Biopsies of insufficient size to count CD3 cells in 4-5 high power fields (HPF; magnification 400x) were excluded. Corresponding areas of noticeable HLA-G expression on tumor cells where quantified only if HLA-G expression was clearly separable between EwS and infiltrating lymphocytes. HLA-G^pos^ areas with strong infiltration, bleeding and necrosis as well as intratumoral stroma where excluded. A 200x magnification was used for all images.

### CAR constructs

All G_D2_-specific CARs contain the single-chain antibody domain (scFv) of the monoclonal antibody (mAb) 14.G2a [48]. The CARs containing CD28 and 2B4 costimulatory domains were previously described [49, 50]. To generate GD2-BBζ, we replaced the transmembrane domain with the one derived from CD28 and exchanged the gene fragment encoding for the cytoplasmic 2B4 domain by the respective signaling domain of 4-1BB. The CAR gene was codon-optimized and subcloned into the *AgeI* and *XhoI* sites of the retroviral vector SFG. Generation of stable retroviral producer cell lines and production of recombinant retrovirus for transduction of T cells was performed as described [48, 50].

### Mouse tissues

Murine tumor xenografts were obtained from previous mouse experiments approved by the animal care committee of the local government (Bezirksregierung Muenster, Az. 87-51.04.2010.A117). For s.c. tumor growth, 2 × 10^6^ VH-64 cells had been injected s.c. into the right flanks of NSG mice. Upon a tumor volume of 200-300 mm^3^, 3 consecutive i.v. doses of T cells gene-modified to express the G_D2_-specific CAR GD2-BBζ had been given (days 2, 9, 15). TC-32 cells had been given i.p. at 0.5-3.0 × 10^6^cells/mouse, followed by adoptive transfer of CAR T cells on days 8, 9, 10 and 11 after tumor cell injection. 20,000 IU recombinant human interleukin-2 (rhIL-2) had been given along with the T cells and again starting on day 15 i.p. twice weekly. Control mice had been treated with phosphate-buffered saline (PBS) with and without IL-2. Fifteen days post therapy, mice had been sacrificed and the tumors were preserved for subsequent histopathological analysis.

### HLA-G upregulation assay

EwS cells from lines VH-64 or TC-32 were seeded at 1 × 10^6^ cells per well in 2 mL medium into wells of a collagen-coated tissue culture 6-well-plate and grown to 100% confluence for 24 hours at 37°C, 5% CO_2_. On day 2, the medium was removed, and 3.7 × 10^5^ non-transduced or GD2-BBζ CAR transduced T cells, thawn on the previous day, were added to each well in 1.5 mL of medium containing 500 IU rhIL-2/mL. After coincubation for 48 hours, the supernatants were harvested, filtrated and added to newly cultured EwS monolayers seeded at 0.25 × 10^6^ cells in 25 cm^2^ collagen-coated flasks. Supernatants from T cell cultures in the absence of EwS monolayers were used in comparison. The T cells from each well were washed by centrifugation, resuspended in 1.5 mL fresh medium with 500 IU rhIL-2/mL, and added to newly grown EwS monolayers in 6-well-plates as described above for additional 72 hours. The supernatants were again harvested, filtrated and used to replace the medium of the EwS cells grown in the culture flasks. After additional 72 hours, tumor cells from the culture flasks were harvested, and HLA-G protein was detected by Western blot as described below.

### ELISA quantification of HLA-G secretion

A commercially available HLA-G kit (Exbio, RD194070100R) was used to quantify HLA-G in cell culture supernatants. Samples were incubated cell free in triplicates for a minimum of 16 hours. Supernatants from JEG-3 and K-562 cells served as positive and negative controls, respectively. HLA-G (U/ml) was quantified according to measurement of the incorporated calibrator.

### Western blot analysis

Cells were homogenized in 100 µL ice-cold RIPA-buffer (0.1% DTT (Sigma-Aldrich, 43815-1G) with fresh protease inhibitor cocktail (Roche, 11873580001), shortly fractured in liquid nitrogen, thawed on ice and then clarified by spinning for 15 minutes at 4°C and 20,000xg. The protein containing supernatant was separated by electrophoresis in an SDS/10% polyacrylamide gel and then electroblotted onto a nitrocellulose membrane (Biorad). Blocking was done in TBST-buffer (5% nonfat dry milk) for 1 hour, followed by incubation with mAb 4H84 diluted 1:1,000 in TBST (5% BSA) for 12 hours at 4°C. After washing, the membrane was incubated with HRP-linked anti-mouse IgG whole Ab (GE Healthcare, NA931) 1:2,000 in TBST 5% milk for 1 hour at RT, followed by treatment with enhanced chemiluminescence reagent (ECL Plus Western Blotting Detection Systems, GE Healthcare) and exposed to Hyperfilm ECL film (GE Healthcare) for 1 minute. Equal protein loading was determined by ß-Actin detection. The membrane was washed with 20 mL TBST buffer for 10 minutes at RT, followed by incubation with 7 mL Restore™ Plus WB stripping buffer (46430, Thermo Scientific) for 15 minutes at RT. After washing twice in 20 mL TBST buffer for 10 minutes at RT, followed by blocking with TBST (5% nonfat dry milk) for 1 hour. β-Actin antibody (4967, Cell Signaling) was diluted 1:5.000 in TBST (5% BSA) for 12 hours at 4°C. After washing, the membrane was incubated with HRP-linked anti-rabbit Antibody (donkey, NA934V, GE Healthcare) 1:2.000 in TBST 5% milk for 1 hour at RT, followed by detection as described above.

### Statistical analysis

Statistical analyses of patient characteristics were performed using IBM SPSS Statistics, Version 23.0 for Windows (IBM Corporation, Somers, NY, USA) and SAS software, Version 9.4 (SAS Institute Inc., Cary, NC, USA), while experimental data was analyzed with Sigmablot Version 11.0. Description of categorical variables was done by reporting absolute and relative frequencies. Group comparisons were performed using Fisher’s exact test to adjust for the relatively small number of evaluable patients. For continuous variables median and range were reported and compared between groups by using non-parametric Mann-Whitney *U* test. Inferential statistics are intended to be exploratory (hypotheses generating), not confirmatory, and are interpreted accordingly. I.e., *p*-values are interpreted in Fisher’s sense, representing the metric weight of evidence against the respective null hypothesis of no effect. Neither a global significance level nor local levels are determined. *P*-values are considered noticeable in case *p* < = 0.05 and highly noticeable in case *p* < = 0.01.

## SUPPLEMENTARY MATERIALS FIGURE



## Abbreviations

BM: Bone marrow
CAR: Chimeric antigen receptor
CD: Cluster of differentiation
EwS: Ewing sarcoma
FFPE: Formalin-fixed paraffin-embedded
HLA: Human Leukocyte Antigen
HPF: High power field
IFN-γ: Interferon-γ
IL-2: Interleukin 2
ILT: Immunoglobulin-like transcript
i.p.: intraperitoneal
KIR: Killer-cell immunoglobulin-like receptors
MHC: Major Histocompatibility Complex
MSC: Mesenchymal stroma cells
NK cell: Natural killer cell
NSG mice: NOD scid gamma mice
PB: Peripheral blood
PBMC: Peripheral blood mononuclear cells
PD-L1: Programmed death ligand 1
PD-1: Programmed cell death protein 1
rhIL-2: recombinant human IL-2
s.c.: subcutaneous
Treg cell: T cell with regulatory phenotype
