# Supplementary material for: T cell infiltration into Ewing sarcomas is associated with local expression of immune-inhibitory HLA-G
Source: Oncotarget. 2017 Dec 22;9(5):6536–49. doi: 10.18632/oncotarget.23815 (PMC5814230; doi:10.18632/oncotarget.23815)
Supplement: Supplementary file 1 [file oncotarget-09-6536-s001.pdf]

T cell infiltration into Ewing sarcomas is associated with local expression of immune-inhibitory HLA-G

SUPPLEMENTARY MATERIALS

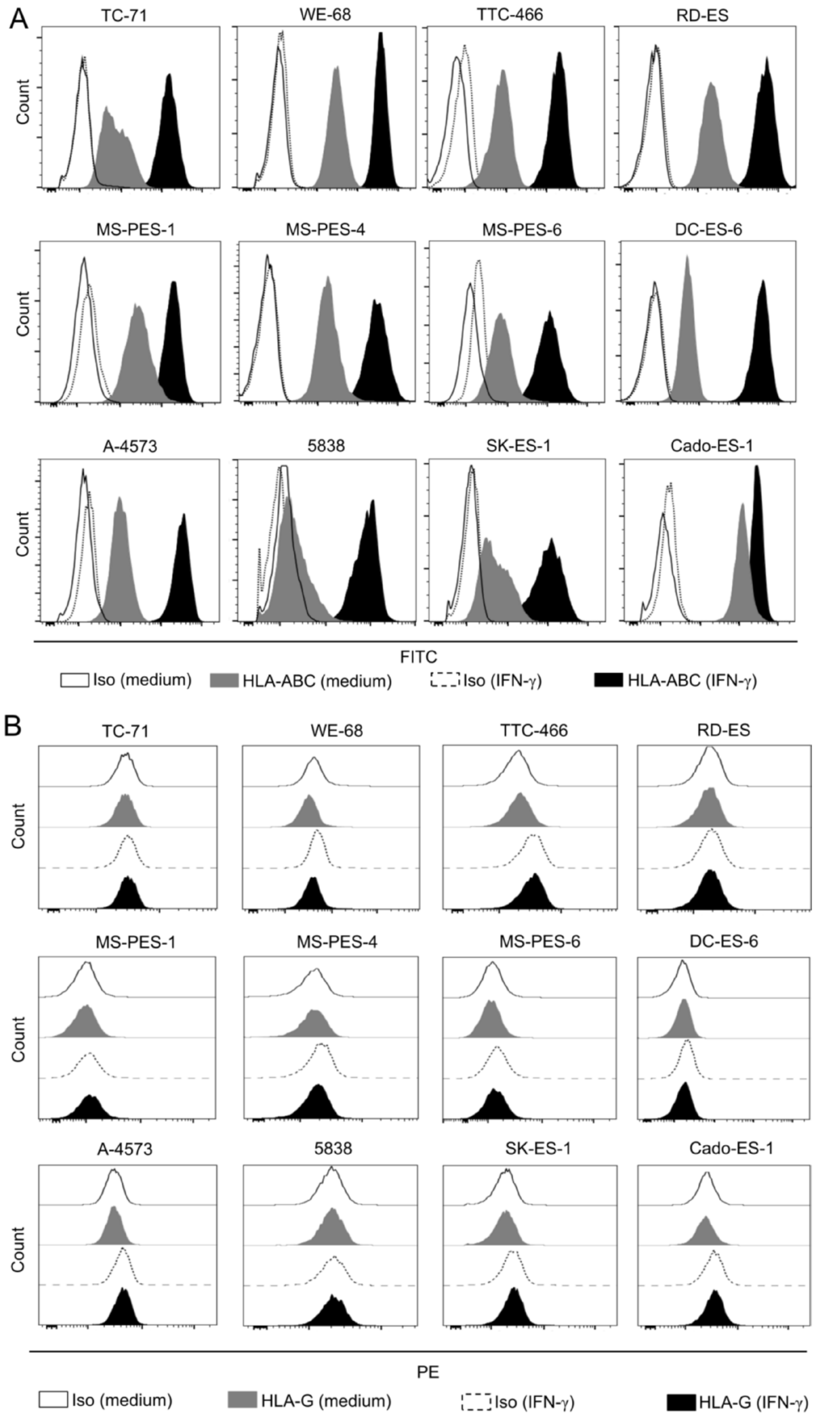

Supplementary Figure 1: HLA-ABC (**A**) and HLA-G (**B**) expression in EwS cells with and without stimulation with IFN-γ (500 U/ml) for 48 hours by flow cytometry (2 experiments).
